# Using supervised learning to develop BaRAD, a 40-year monthly bias-adjusted global gridded radiation dataset

**DOI:** 10.1038/s41597-021-01016-4

**Published:** 2021-09-15

**Authors:** T. C. Chakraborty, Xuhui Lee

**Affiliations:** School of the Environment, Yale University, New Haven, CT 06520 USA

**Keywords:** Atmospheric science, Hydrology

## Abstract

Diffuse solar radiation is an important, but understudied, component of the Earth’s surface radiation budget, with most global climate models not archiving this variable and a dearth of ground-based observations. Here, we describe the development of a global 40-year (1980–2019) monthly database of total shortwave radiation, including its diffuse and direct beam components, called BaRAD (Bias-adjusted RADiation dataset). The dataset is based on a random forest algorithm trained using Global Energy Balance Archive (GEBA) observations and applied to the Modern-Era Retrospective analysis for Research and Applications, Version 2 (MERRA-2) dataset at the native MERRA-2 resolution (0.5° by 0.625°). The dataset preserves seasonal, latitudinal, and long-term trends in the MERRA-2 data, but with reduced biases than MERRA-2. The mean bias error is close to 0 (root mean square error = 10.1 W m^−2^) for diffuse radiation and −0.2 W m^−2^ (root mean square error = 19.2 W m^−2^) for the total incoming shortwave radiation at the surface. Studies on atmosphere-biosphere interactions, especially those on the diffuse radiation fertilization effect, can benefit from this dataset.

## Abstract (summary)

**Table:** 

Measurement(s)	radiation components at the surface
Technology Type(s)	machine learning
Factor Type(s)	radiation
Sample Characteristic - Environment	climate
Sample Characteristic - Location	global

Machine-accessible metadata file describing the reported data: 10.6084/m9.figshare.15090462

## Background & Summary

The Earth’s climate is driven by solar (shortwave) radiation and its interactions with the different components of the Earth system. The shortwave radiation is attenuated by scattering and absorption by atmospheric aerosols, clouds, and gases, with the remaining portion reaching the Earth’s surface as direct beam radiation (*K*_↓,b_). A portion of the scattered radiation also reaches the surface, which deviates from its original path and is known as diffuse radiation (*K*_↓,d_). The sum of *K*_↓,b_ and *K*_↓,d_, or the total incident shortwave radiation at the surface (*K*_↓_), influences local weather and climate, the hydrological cycle, and the carbon budget. There is also strong scientific interest in *K*_↓,d_ because a high diffuse fraction can increase agricultural and ecosystem productivity and enhance the terrestrial water flux to the atmosphere through increased photosynthesis in normally shaded parts of the plant canopy, a phenomenon known as the diffuse radiation fertilization effect^1–3^.

Current Earth System Models (ESMs) generally overestimate *K*_↓_ compared to observations, primarily due to errors associated with parameterizations of clouds and aerosols^4–7^. This overestimation would cause artificial surface warming, with undesired consequences on atmosphere-biosphere interactions^8,9^. Although similar evaluations of ESM *K*_↓,d_ are not available, large differences are reported for *K*_↓,d_ between reanalysis datasets and observations^10^. The bias in *K*_↓,d_ in these gridded datasets is not consistent in direction, unlike that for *K*_↓_. Such biases may contribute to uncertainties in modelling surface energy and carbon budgets and impact optimum placement of concentrating solar power systems^11,12^.

Several previous studies have examined the biases in modeled *K*_↓_ using the clearness index (*k*_t_). This index, defined as the ratio between surface incident and extraterrestrial radiation, captures the combined impact of aerosols, clouds, and gases on atmospheric transmittance on solar radiation^13–15^. These atmospheric constituents attenuate solar radiation as it moves through the atmospheric column. Although *k*_t_, a measure of the total light extinction, directly affects *K*_↓,b_ and therefore exerts a strong control on *K*_↓_, it is only tangentially related to *K*_↓,d_. It is known that *K*_↓,d_ is primarily controlled by the abundance of scattering agents in the atmosphere, as well as their degree of forward scattering^16^. An atmospheric scattering agent that reduces *K*_↓,b_ may actually increase *K*_↓,d_. Thus, a new approach is required to correct biases in *K*_↓,d_.

In recent years, machine learning algorithms have been used to reduce biases in radiation fields derived from reanalysis products or derive the fields from satellite observations^17–22^. By training against observed data, these algorithms can capture previously unknown relationships between actual and gridded variables, generally leading to improvements over traditional parametric and multi-ensemble averaging techniques^22^. However, the majority of these algorithms have been implemented at the regional scale, particularly over China, Europe, and the US, with a focus on the total *K*_↓_. For reasons briefly described above, it is also important to develop a generalizable bias-correction algorithm for *K*_↓,d_. Of note, a recent study developed a global hourly *K*_↓,d_ dataset using a random forest algorithm on satellite retrievals from the Earth Polychromatic Imaging Camera (EPIC)^21^, although this focused on a short period from June 2015 to June 2019. A gridded data product after proper bias correction is especially welcome for tropical regions where *K*_↓,d_ measurements are rare but the diffuse fertilization effect is strong due to high vegetation densities^3,23^.

In this paper, we describe the development of a new dataset of monthly gridded radiation fields, including *K*_↓,_ *K*_↓,b_, and *K*_↓,d_, from 1980 to 2019, which can be explored through this web application: https://yceo.users.earthengine.app/view/barad. We attempt to improve historical global gridded estimates of *K*_↓,d._ through three major steps:

1. Examine the control of *k*_t_ on biases in *K*_↓,_ *K*_↓,b,_ and *K*_↓,d_ separately
2. Test bias-correction algorithms for *K*_↓_ and *K*_↓,d_, including a method based on *k*_t_, a multiple linear regression (MLR) and a random forest (RF) model
3. Implement the best performing bias-correction algorithm to create a global 40-year Bias-adjusted RADiation dataset, or BaRAD.

## Methods

### Reanalysis data

The gridded data reported here is based on the Modern-Era Retrospective analysis for Research and Applications, version 2 (MERRA-2) global reanalysis dataset^24^. MERRA-2 improves upon the original MERRA dataset in several ways. It adds an extensive aerosol assimilation by using bias-adjusted aerosol optical depth (AOD) from satellite observations^24^. Unlike MERRA, MERRA-2 uses observed precipitation to force the land-surface model^25^. It uses a newer version of the Goddard Earth Observing System (GEOS-5) and assimilates newer satellite observations of aerosols, clouds, and precipitation^26^. MERRA-2 is available from 1980 to present day at a grid resolution of 0.5° latitude and 0.625° longitude. The variables we wish to correct are monthly mean *K*_↓_ and *K*_↓,d_ using predictors that physically control transmitted radiation. They include estimates of atmospheric clouds and aerosols, as well information about the position of the Sun, which controls energy input to the atmospheric column.

### Ground-Based observations for training and validation

We used the Global Energy Balance Archive (GEBA) for training and validation of bias correction algorithms. GEBA is a comprehensive observational data repository of the components of the Earth’s surface energy budget^27^. The latest version of the database has roughly 2500 unique stations^28^. Here, we used the monthly mean *K*_↓_ and *K*_↓,d_ stored in the database. The data were screened with several quality control steps. We only selected the monthly mean values lower than 600 W m^−2^ for *K*_↓_ and 250 W m^−2^ for *K*_↓,d_. Cases where the ratio of modeled to observed monthly means exceed 5 were ignored. Finally, only sites with all 12 months of available data were selected to avoid biased representation across seasons. After these data screening steps, we obtained 935 unique sites with 134541 site-months of data for *K*_↓_ and 290 unique sites with 28880 site-months for *K*_↓,d_ between 1980 and 2017 (Fig. S1). Monthly mean *K*_↓,b_ was computed as the difference between *K*_↓_ and *K*_↓,d_.

### Bias-Correction algorithms

We tested three bias correction algorithms, including a technique based on clearness index and two data-driven algorithms. Several studies have used clearness index ${k}_{t}$ as a threshold for designating sky condition or for estimating *K*_↓_^13,29,30^. In Zhao *et al*.^29^, the bias in *K*_↓_ (*b*_m_) is related to *k*_t_ in a linear fashion:

1 $${b}_{{\rm{m}}=}{b}_{0.}{k}_{{\rm{t}}+}{b}_{1}$$

Here *b*_0_ is the sensitivity of *b*_m_ to *k*_t_, and *b*_1_ is the model bias ratio under completely cloudy conditions. In their study, *b*_m_ is given as

2 $${b}_{{\rm{m}}}=\frac{{K}_{{\rm{R}}}-{K}_{{\rm{O}}}}{{K}_{{\rm{R}}}}$$

where *K*_R_ and *K*_O_ are modeled and observed values, respectively. Clearness index is given by

3 $${k}_{{\rm{t}}}=\frac{{K}_{\downarrow {\rm{,o}}}}{{K}_{{\rm{TOA}}}}$$

where *K*_TOA_ is the extra-terrestrial radiation at the top of the atmosphere and *K*_↓,O_ is the observed *K*_↓_ value. Their method subsequently also accounted for site elevation *H* through a somewhat arbitrarily chosen quadratic fitting function. Here, we used a multi-linear regression (MLR) model, which the authors^29^ note would yield similar results, as a function of *k*_t_, *H*, and *K*_↓,R_, the *K*_↓_ from the reanalysis without correction, to correct *K*_↓_

4 $${K}_{\downarrow }={\beta }_{0}{K}_{\downarrow ,{\rm{R}}}+{\beta }_{1}\;{k}_{{\rm{t}}}+{\beta }_{2}\;H+{\beta }_{3}$$

where *β*_0_, *β*_1_, *β*_2_, and *β*_3_ are empirical coefficients. A linear model of the same form was also used to correct *K*_↓,d_. Since *k*_t_ involves observed *K*_↓_ (Eq. 1), Eq. 4 cannot be used to correct biases in gridded data when observations are not available. Thus, we considered two variations of this algorithm, one using observed *K*_↓_ (*K*_↓,O_) and site elevation to calculate clearness index, called the *k*_t_,_O_ model, and the other using grid-averaged terrain elevation (*H*_R_) and the clearness index calculated from modeled *K*_↓_ (*K*_↓,R_), given by:

5 $${k}_{{\rm{t}}}=\frac{{K}_{\downarrow {\rm{,R}}}}{{K}_{{\rm{TOA}}}}$$

which we call the *k*_t_,_R_ model.

The second algorithm, another MLR model, expresses the dependent variable as a linear combination of predictors. In the case of *K*_↓_, it takes the following form

6 $${K}_{\downarrow ,{\rm{O}}}={\beta }_{0}{K}_{\downarrow ,{\rm{R}}}+{\beta }_{1}\;{\rm{SAOD}}+{\beta }_{2}\;{\rm{AAOD}}+{\beta }_{3}\;{\rm{COD}}+{\beta }_{4}\;{\rm{CF}}+{\beta }_{5}\;{\theta }_{z}+{\beta }_{6}\;{H}_{{\rm{R}}}+{\beta }_{7}$$

where *β*_0_ to *β*_7_ are regression coefficients, *K*_↓,O_ is the observed (or bias corrected) *K*_↓_, *K*_↓,R_ is the *K*_↓_ from the reanalysis without correction, SAOD is scattering aerosol optical depth (AOD), AAOD is absorption AOD, COD is cloud optical depth, CF is cloud fraction, and *θ*_z_ is the monthly mean zenith angle – the angle between the sun and the vertical direction – estimated from the hourly values. The MLR procedure with the same set of predictors was also applied to *K*_↓,d_. These predictors (summarized in Table S1) provide strong physical constraints on atmospheric radiative transfer^31^, with both COD and AOD being direct measures of light extinction along the atmospheric column. Correlation matrices for the features selected show that other than for *θ*_z_ and the radiation field (*K*_R_) and AAOD and SAOD, the correlations coefficients between the features are generally smaller than 0.75. Although AAOD and SAOD are strongly correlated, the separation of AOD into SAOD and AAOD is more important for *K*_↓,d_ than for *K*_↓_ since while absorption of solar radiation by aerosols would reduce both *K*_↓,d_ and *K*_↓,b,_ forward scattering would reduce *K*_↓,b_ and increase *K*_↓,d_. With the intent of developing a generalized algorithm, one regression is used for the entire dataset. Since *θ*_z_ strongly controls the optical thickness of the atmosphere even for clear-sky conditions^32^ and is one of the predictors, seasonal and latitudinal effects are accounted for to some extent. The algorithm was implemented using the stats package on the R programming language.

The third algorithm is a random forest (RF) regression technique^33^. Unlike the MLR models, the RF regression does not assume a standard linear structure of the relationship; instead it derives the relationship from the training data using an ensemble of decision trees. This relationship (for the total incoming radiation) can be expressed in a generic form as:

7 $${K}_{\downarrow {\rm{,O}}}=f\left({K}_{\downarrow ,{\rm{R}},}{\rm{SAOD,}}\,{\rm{AAOD}},\;{\rm{COD,}}\;{\rm{CF,}}\;{\theta }_{z},{H}_{{\rm{R}}}\right)$$

This random forest regression was implemented using the R Random Forest package. The default minimum size of terminal nodes (5) was used, but the maximum number of trees to generate was set to 2000. In most folds, the models converged before reaching this limit. As per the default parameters of the package, each tree is trained on 63.2% of the training data with 2 predictor variables chosen at random to split the nodes. Trees were allowed to grow fully rather than be pruned.

We used a 10-fold cross-validation technique to evaluation the performance of these algorithms. The entire GEBA dataset was randomly partitioned into 10 equal subsets. One of the ten subsets was used for validation and the other nine for training. The process was repeated 10 times. The accuracy was quantified using the coefficient of determination (*r*^2^), the root mean square error (RMSE), and the mean bias error (MBE). Cross-validation is desired for the RF algorithm because it is prone to overfitting and using multiple folds allows us to examine the consistency of the results across different training/validation splits. The two linear models (Eqs. 4 and 6) are not prone to overfitting. However, because they are sensitive to outliers, cross-validation was also done to estimate the influence of the training data selection on their performance.

The final data product (BaRAD) consists of monthly *K*_↓,_ *K*_↓,b,_ and *K*_↓,d_ corrected with the best performing algorithm, defined as the one with minimum RMSE and highest *r*^2^ in the consolidated validated data at the native MERRA-2 resolution. Here the algorithm was trained on the whole quality screened GEBA dataset.

### Clearness index as a predictor of bias

Zhao *et al*.^29^ found systematic overestimation of *K*_↓_ in two reanalysis datasets. To correct these model biases, they utilized the empirical relationship between the sensitivity of *b*_m_ to the observed *k*_t_. Here the sensitivity is the slope of the linear regression between *b*_m_ and *k*_t,O_. To illustrate how this sensitivity varies between *K*_↓_, *K*_↓,d_, and *K*_↓,b_, we separately examined the associations between *b*_m_ and *k*_t,O_.

Unsurprisingly, *b*_m_ for *K*_↓_ and *k*_t,O_ are negatively correlated, both overall and for the common sites (Fig. S2b and d). Here the common sites are those with simultaneous measurements of *K*_↓_ and *K*_↓,d_. The sensitivity of *b*_m_ to *k*_t,O_ is −0.76 for all sites and −0.8 for common sites, which are very close to the value of −0.82 found by Zhao *et al*.^29^ for MERRA in North America. Similarly, *b*_m_ for *K*_↓,b_ is also negatively correlated with *k*_t,O_, with the sensitivity being higher in magnitude (−1.23; Fig. S2c) than that for *K*_↓_, suggesting that total atmospheric transmittance has a stronger effect on the biases in *K*_↓,b_ than on the biases in *K*_↓_. For *K*_↓,d_, the sensitivity of *b*_m_ to *k*_t,O_ is strong (−0.89; Fig. S2a), but the variability in the bias is not explained well by it (*r*^2^ = 0.15). Overall, the coefficient of determination (*r*^2^) is highest for *K*_↓,b_ and smallest for *K*_↓,d_, indicating that clearness index is a poor predictor of model bias in *K*_↓,d_.

It is also important to note the intercept of the equations shown in Fig. S2. This intercept represents the *b*_m_ for a completely non-transmissive atmosphere (i.e. when *k*_t,O_  = ﻿0). For both *K*_↓_ and *K*_↓,b_, this value is positive (0.96 for *K*_↓,b_; 0.5 to 0.53 for *K*_↓_). This implies that the reanalysis overestimates *K*_↓_ under non-overcast skies, and its estimates improve for clearer conditions. On the other hand, the intercept for the regression line between the *b*_m_ for *K*_↓,d_ and *k*_t,O_ is close to zero and the slope is negative, suggesting that MERRA-2 *K*_↓,d_ is underestimated even under completely clear conditions.

### Comparing bias-correction algorithms

**Fig. 1:**
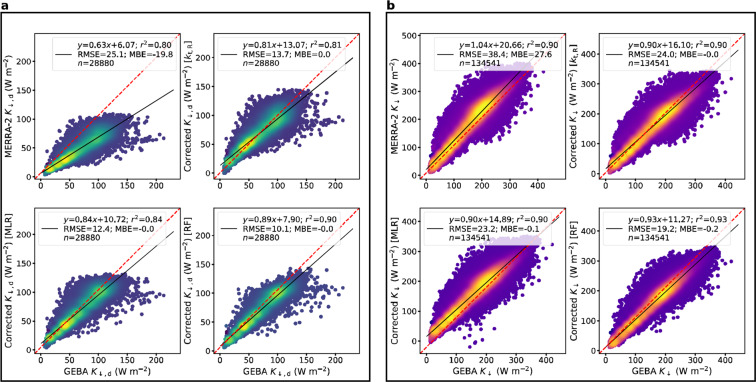
Comparison of original and bias-adjusted MERRA-2 data with GEBA observations. (**a**) monthly mean diffuse radiation (*K*_↓,d_) and (**b**) total shortwave radiation (*K*_↓,_) from MERRA-2 as well as the bias-adjusted estimates from the *k*_t,R_, MLR, and RF models. For the bias-adjusted estimates, the consolidated validation data from all 10 folds are shown. The red dashed lines represent the 1:1 relationship. Color indicates data density and the statistical summaries of the evaluations are noted.

**Fig. 2:**
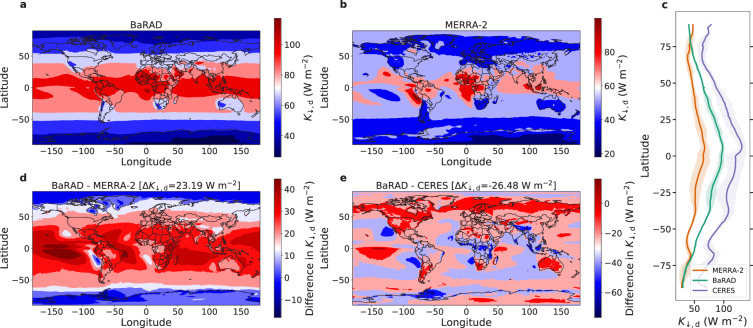
Spatial and latitudinal variability in diffuse radiation. Global pattern of diffuse radiation (*K*_↓,d_) in (**a**) the BaRAD product, (**b**) the MERRA-2 dataset, and (**c**) the CERES dataset. The grid-wise difference between BaRAD and (**e**) MERRA-2 and (**f**) CERES are also shown. Sub-figure (**b**) shows the mean latitudinal variability of *K*_↓,d_ in all three products. The shaded areas represent the standard deviation. The area-weighted mean difference in *K*_↓,d_ (Δ*K*_↓,d_) between the BaRAD data and the MERRA-2 and CERES products, respectively, are shown at the top of sub-figures (**d**) and (**e**), respectively.

Figure 1 shows the comparison of the original MERRA-2 and bias-adjusted values with the GEBA observations. MERRA-2 underestimates *K*_↓,d_ (MBE = −19.8 W m^−2^; Fig. 2a) and overestimates *K*_↓_ (MBE = 27.6 19.8 W m^−2^; Fig. 2b). Consistent with the *K*_↓_ overestimation, the modeled clearness index *k*_t_,_R_ (0.54 ± 0.11) is higher than the observed index *k*_t_,_O_ (0.45 ± 0.12). This increased transmissivity may be caused by underestimation of both clouds and aerosols, although clouds probably play a greater role since MERRA-2 has assimilated observations of AOD. Although an underestimation in clouds would also explain the underestimation in *K*_↓,d_, the intercept of the equation in Fig. S2a (see previous subsection) suggests that clouds are not the only factor.

All the three algorithms reduce the MBE and RMSE of *K*_↓,_ *K*_↓,b,_ and *K*_↓,d_ in comparison to the original MERRA-2 values. The RF model performs the best overall, minimizing the RMSE and maximizing *r*^2^ for both *K*_↓_ (RMSE = 19.2 W m^−2^; *r*^2^ = 0.93) and *K*_↓,d_ (RMSE = 10.1 W m^−2^; *r*^2^ = 0.90). The Taylor diagrams for the composite validation dataset, along with the results for both the *k*_t_,_O_ and *k*_t_,_R_ models, are in the supplementary information (Fig. S3). The RF model consistently outperforms the others for every fold (with one exception; see below). For *K*_↓,d_, the MLR model is not as good as the RF model but is better than the *k*_t_,_R_ and *k*_t_,_O_ models (Fig. S3a). For *K*_↓_, the *k*_t_,_O_ model performs slightly better than the RF model (Fig. S3b), which makes sense since *k*_t_,_O_ includes the observed *K*_↓_, and thus this model, not useable to correct global datasets, is not shown in Fig. 1. That the *k*_t_,_O_ model outperforms the other models for *K*_↓_ but not for *K*_↓,d_ confirms our hypothesis that the *k*_t_ model is not appropriate to address biases in *K*_↓,d_. Similar to the *k*_t_,_R_ MLR model (Eq. 4), we also train a RF model with ${k}_{t}$ derived from MERRA-2 as one of the features. Consistent with the results of the *k*_t_,_O_ MLR and *k*_t_,_R_ MLR models, the *k*_t_,_R_ RF model cannot beat the performance of the RF model using MERRA-2 features (Eq. 7).

Physically, the monthly average radiation components cannot be negative. However, both the *k*_t_,_R_ and MLR models predict a small fraction of negative values for *K*_↓_ (0.15% for *k*_t_,_R_ and 0.10% for MLR) and *K*_↓,d_ (0.24% for *k*_t_,_R_ and 0.01% for MLR). One can account for this by setting these negative values to zero after correction. However, this imposed physical constraint is not required for the RF corrected values. We also test whether the distribution of predicted values by the RF model is statistically different from those predicted by the other models using paired Wilcoxon Sign Rank Tests^34^. In all cases, except compared to the *k*_t_,_O_ model for *K*_↓_ (p-value = 0.58; supporting the null hypothesis of no difference), there are statistically significant differences between the tested algorithms. This is further evidence of the usefulness of *k*_t_ based models for *K*_↓_ but not *K*_↓,d_.

MLR and RF use the same gridded variables as predictors. Fig. S5 presents the feature importance of each variable. For the RF model, a feature importance is the increase in mean square error (MSE) of the predicted values if the same variable is removed from the model. A higher increase in MSE indicates that the variable is more important to the performance of the RF model. Although there are several established methods for interpretating the relative importance of variables for MLR models, for ease of comparison, we use a model-agnostic permutation method similar to the one used for the RF model using the iml package for the R programming language. For the RF model, the two best predictors are different for *K*_↓,d_ and for *K*_↓_. For *K*_↓,d_, COD and CF have the highest importance scores (224.4 ± 3.3% for COD; 138.2 ± 3.1% for CF; Fig. S5c), and for *K*_↓_, AAOD and SAOD have the highest importance scores (223.6 ± 16.5% for AAOD; 206.5 ± 7.5% for SAOD; Fig. S5d). In contrast, the radiation field is the most important variable in the MLR models for both *K*_↓_ (451.2 ± 1.7%) and *K*_↓,d_ (276.5 ± 1.1%; Figs. S5a and S5b). These differences are expected since the model architectures are also different, with the MLR model assuming linear relationships between the output and the input features and the RF model also accounting for non-linear interactions.

### The BaRAD dataset

Based on our cross-validation results, we choose the RF model to adjust the biases in the MERRA-2 *K*_↓_ and *K*_↓,d._ We re-trained the model twice, one for *K*_↓_ and the other for *K*_↓,d_, using the same predictors and all available quality screened GEBA observations (instead of random training subsets of it as done during the cross-validation phase). The trained model was used to bias-adjust the corresponding gridded monthly MERRA-2 fields from 1980 to 2019. The bias-adjusted dataset is referred to as BaRAD. The final BaRAD data deposited in the public archive has gone through two additional post-correction adjustments. First, because of lack of training data in polar regions, there exist a few positive values at some polar grids during polar nights; these positive values constitute 6.5% of the entire dataset for *K*_↓_. Here we have forced the bias-adjusted *K*_↓_ and *K*_↓,d_ to zero when the corresponding MERRA-2 values are zero in those grids. Second, since *K*_↓,d_ and *K*_↓_ were trained separately, there is a small fraction of gridded data (less than 0.5%) where *K*_↓,d_ exceeds *K*_↓_, which is physically impossible. For these cases, we have set the *K*_↓,d_ value equal to *K*_↓_.

## Data Records

The BaRAD dataset is available in netCDF format and includes the monthly values of *K*_↓_ (variable name: K_down), *K*_↓,d_ (variable name: K_diff), and *K*_↓,b_ (variable name: K_dir) starting from January, 1980^35^. Separate netCDF files are generated for each year from 1980 to 2019. All variables have the unit of W m^−2^ and are available at the MERRA-2 native resolution of 0.5° by 0.625°. The BaRAD dataset generated in this study is available in this GitHub repository: https://github.com/TC25/BaRAD/tree/main/BaRAD_Dataset and also through PANGAEA (10.1594/PANGAEA.932924)^35^. The training data are also available in the main GitHub repository.

## Technical Validation

### Comparison of BaRAD dataset with other gridded data products

In Figs. 2, S6, and 3, we compare the spatial, zonal, and seasonal patterns in the BaRAD dataset with the original MERRA-2 dataset. We also compare these patterns with the latest version of the Clouds and the Earth’s Radiant Energy System (CERES) surface radiation product^36^. The CERES dataset provides satellite-based estimates of the Earth’s radiative budget (from the surface to the top of the atmosphere) and clouds. The data are available globally at 1° by 1° resolution from 2000 onwards. The latest version (CERES_SYN1deg_Ed4.1) of the dataset includes monthly estimates of both *K*_↓,d_ and *K*_↓_.

Although the three datasets show broadly similar latitudinal (Figs. 2c and S6c) and spatial patterns (Figs. 2d,e, S6d, and S6e), *K*_↓,d_ in the BaRAD dataset is higher than in MERRA-2 over the Sahara and India and higher than the CERES data over Australia. For *K*_↓_, BaRAD shows a lower value than both MERRA-2 and CERES over the tropical region. Figures 2 and S6 also show the mean area-weighted difference (Δ*K*_↓_ and Δ*K*_↓,d_) between the BaRAD data and the MERRA-2 and CERES products, respectively. The global mean *K*_↓_ and *K*_↓,d_ are 167.9 and 75.8 W m^−2^, respectively, according to BaRAD. In comparison, the global mean *K*_↓_ is higher at 185.4 W m^−2^ and 185.9 W m^−2^ according to MERRA-2 and CERES respectively, and the global mean *K*_↓,d_ is lower at 52.6 W m^−2^ according to MERRA-2 and higher at 102.4 W m^−2^ according to CERES.

**Fig. 3:**
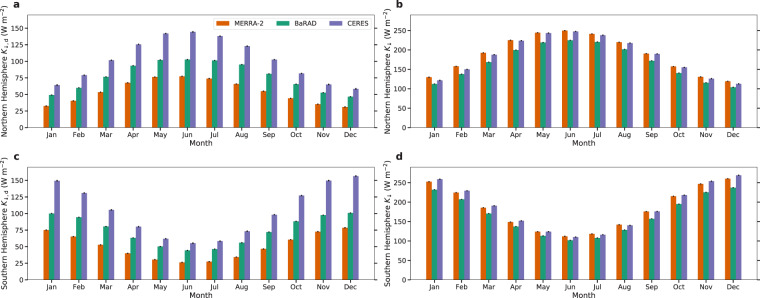
Seasonal variability in all products. Monthly variability in diffuse radiation (*K*_↓,d_) in MERRA-2, BaRAD, and CERES for (**a**) the northern hemisphere and (**c**) the southern hemisphere. Sub-figures (**b**) and (**d**) are the same, but for total shortwave radiation (*K*_↓_). The error bars show the standard errors for each month.

We calculate the seasonal trends of *K*_↓,d_ and *K*_↓_ in the northern and southern hemisphere grids (Fig. 3). Although there are large differences in the magnitude of the three datasets, the seasonal variation is captured by the BaRAD dataset  (when compared to the other two). For instance, the highest northern hemisphere averages are during the boreal summer and the lowest values are during the winter; vice versa for the southern hemisphere. These patterns are evident in all the datasets.

**Fig. 4:**
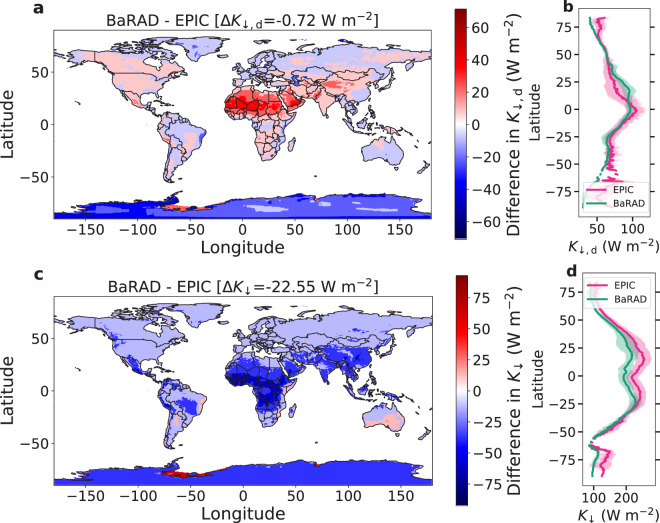
Comparison of spatial and latitudinal variability in total shortwave radiation and diffuse radiation between the BaRAD product and EPIC-derived estimates. Spatial patterns of the grid-wise difference in (**a**) diffuse radiation (*K*_↓,d_) and (**b**) total shortwave radiation (*K*_↓_) over land. Sub-figure (**b**) and (**d**) show the mean latitudinal variability of *K*_↓,d_ and *K*_↓_ over land for the two products. The shaded areas represent the standard deviation. The area-weighted difference in *K*_↓,d_ (Δ*K*_↓,d_) and *K*_↓_ (Δ*K*_↓_) between the BaRAD product and the EPIC-derived dataset are shown at the top of sub-figures (**a**) and (**c**), respectively.

We also compare the BaRAD dataset with the newly developed *K*_↓_ and *K*_↓,d_ datasets from the EPIC measurements between 2016 and 2019^21^. The EPIC instrument housed on the Deep Space Climate Observatory (DSCOVR) satellite, takes narrow band spectral images of the sunlit face of Earth for 10 channels every 60 to 100 min. The dataset generated by Hao *et al*.^21^ is available at 0.1° by 0.1° resolution and is based on a random forest algorithm trained using *in situ* observations and the EPIC-derived variables^37^. Here, we compare the available observations with the BaRAD data for the same period. Although the EPIC-based dataset has several advantages over many existing global estimates of *K*_↓,d_, namely the much higher spatial and temporal (up to hourly) resolution, it is not ideal for studying climatological trends. The EPIC instrument is affected by cloud cover and downtime. Thus, the EPIC data are interrupted by data gaps, with 5.1% of days missing between 2016 and 2019. Moreover, the product is only available over land. We regridded the EPIC-derived data to the native MERRA-2 resolution using a nearest neighbor interpolation and compared the spatial and latitudinal trends in the *K*_↓,d_ and *K*_↓_ with the BaRAD values (Fig. 4). Overall, the global mean *K*_↓,d_ in BaRAD is very close to the EPIC-derived values, with a mean difference of only −0.72 W m^−2^. Greater differences are seen for *K*_↓_ with BaRAD underestimating it by 22.55 W m^−2^. Many of the differences between the two products occur over Africa, as also seen from the latitudinal trends (Fig. 4b,d). It is important to note that the *in situ* observations used in Hao *et al*.^21^ to evaluate the product lacks spatial representation over central Africa, while the GEBA observations are much more frequent here, at least for *K*_↓_ (Fig. S1). For *K*_↓,d_, both GEBA and the datasets used in Hao *et al*.^21^ are sparse, which could explain the low Δ*K*_↓,d_ for this variable.

### Validation against baseline surface radiation sites in the tropics

Given the lack of observations in tropical regions and in the southern hemisphere, we examined how the lack of data in those regions affect the BaRAD results. To do so, we processed minute-level observations from the Baseline Surface Radiation Network (BSRN)^38^ and found two sites with sufficient (more than 8650 h in a year) observations in these data-scarce regions to evaluate the gridded products (namely MERRA-2, BaRAD, and CERES). The observations are from the GOB (Gobabeb, Namib Desert, Namibia at 23.56° S, 15.04° E) and PTR (Petrolina, Brazil at 9.07° S, 40.32° W) stations, shown as black stars in Fig. S1b. For the GOB site, 2013, 2014, and 2015 are the years with sufficient observations, while for the PT site, the years 2010, 2011, and 2014 are chosen. Note that although the GEBA dataset includes several BSRN sites, these two sites (and some others) are not included since they do not have enough observations to reliably compute monthly means.

**Fig. 5:**
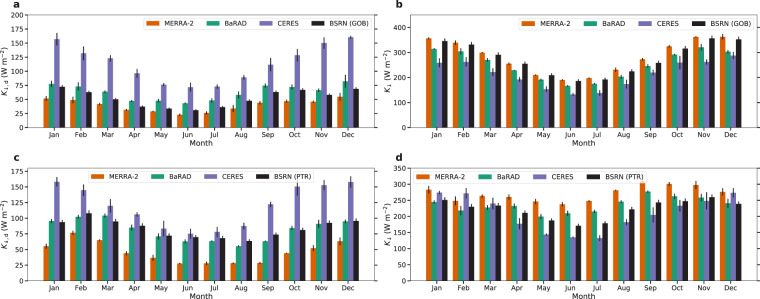
Validation of BaRAD against BSRN observations. Monthly variability in gridded values in MERRA-2, BaRAD, and CERES, and values observed at the GOB (Gobabeb, Namib Desert, Namibia at 23.56° S, 15.04° E) BSRN station for (**a**) diffuse radiation (*K*_↓,d_) and (**b**) total shortwave radiation (*K*_↓_). Sub-figures (**c**) and (**d**) are the same, but for the PTR (Petrolina, Brazil at 9.07° S, 40.32° W) BSRN site. The error bars show the standard errors for each month.

Figure 5 shows the seasonal trends of *K*_↓,d_ and *K*_↓_ from the available BSRN observations, as well as the corresponding monthly composites from MERRA-2, BaRAD, and CERES. For both the stations, the BaRAD data shows less bias (MBE = 10.49 W m^−2^ for GOB; 2.31 W m^−2^ for PTR) than both MERRA-2 (MBE = −12.54 W m^−2^ for GOB; −37.63 W m^−2^ for PTR) and CERES (MBE = 61.76 W m^−2^ for GOB; 36.33 W m^−2^ for PTR) for *K*_↓,d_. CERES overestimates *K*_↓,d_ and MERRA-2 underestimates it compared to observations, which is consistent with the hemispherical results in Fig. 3 and previous estimates. For *K*_↓_, the results are mixed, with BaRAD performing better than CERES but worse than MERRA-2 (MBE = 6.95, −25.17, and −62.97 W m^−2^ for MERRA-2, BaRAD, and CERES, respectively) at the GOB site and better than MERRS-2 and comparable to CERES (MBE = 48.16, 13.21, and −13.17 W m^−2^) at PTR. Note that for the GOB site, there is frequently more missing data at night or early morning than during daytime, which would lead to artificially higher annual *K*_↓_ values than true annual composites, making the comparison with BaRAD seem worse (and vice versa for MERRA-2).

### Long-term trends

Figure S7a–d show the 40-year trend in *K*_↓_ and *K*_↓,d_ in the MERRA-2 and the BaRAD dataset for the two hemispheres. The two datasets show similar trends for *K*_↓_ and *K*_↓,d_, but they are offset by about 20 W m^−2^ for both *K*_↓_ and *K*_↓,d_. More importantly, the BaRAD dataset captures the impacts of the two large volcanic eruptions, El Chichón in 1982 and Mount Pinatubo in 1991, on *K*_↓,d_, particularly in the northern hemisphere (Fig. S7). This is probably because the aerosol, cloud, and radiation fields from the MERRA-2 reanalysis, which is known to capture large volcanic activity^39^, are used to create the BaRAD dataset. For the northern hemisphere, the anomaly in *K*_↓_ from the mean of the previous and subsequent years (1981 and 1983) due to the El Chichón eruption was −1.95 W m^−2^ in MERRA-2 versus −2.81 W m^−2^ in the BaRAD dataset. For the Mount Pinatubo eruption, the *K*_↓_ anomaly was −1.28 W m^−2^ in MERRA-2 versus −1.39 W m^−2^ in the BaRAD dataset. For northern hemisphere *K*_↓,d_, there was an increase by 2.67 W m^−2^ in 1982 compared to the average of the values in 1981 and 1983 in MERRA-2 and 2.13 W m^−2^ for BaRAD. Similarly, in 1991, the northern hemisphere *K*_↓,d_ was higher by 1.75 W m^−2^ compared to 1990 and 1992 in MERRA-2 versus 1.14 W m^−2^ in BaRAD.

**Fig. 6:**
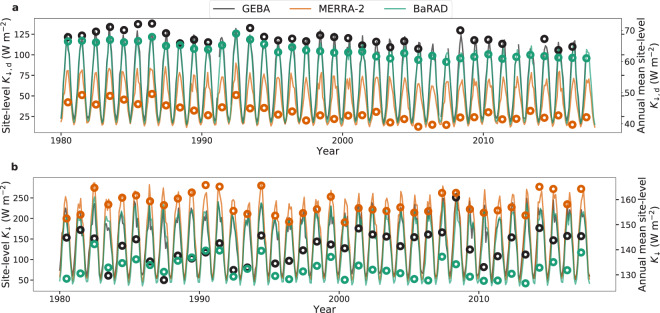
Long-term trends at site scale. Long-term trends in (**a**) diffuse radiation (*K*_↓,d_) and (**b**) total shortwave radiation (*K*_↓_) for GEBA sites with longest archival history, along with corresponding gridded values from MERRA-2 and BaRAD. For *K*_↓,d_, the site with the longest archival history is located in Würzburg, Germany (49.77° N, 9.97° E) and the site with the longest archival history of *K*_↓_ is in Sapporo, Japan (43.05° N, 141.33° E). The monthly values are plotted on the left y-axes as lines and the annual averages (plotted as circles) are on the right y-axes.

Figure 6a, b are two examples of site-level comparison with observations made at Sapporo, Japan (43.05° N, 141.33° E for *K*_↓_) and Würzburg, Germany (49.77° N, 9.97° E for *K*_↓,d_). These two sites are chosen because they have the longest data availability. The BaRAD dataset replicates both the magnitude and long-term variability of the site observations (*r*^2^ = 0.99 and MBE = −3.65 W m^−2^ for *K*_↓,d_; *r*^2^ = 0.97 and MBE = −8.64 W m^−2^ for *K*_↓_). On the other hand, MERRA-2 captures the variability (*r*^2^ = 0.98 for *K*_↓,d_; 0.97 for *K*_↓_), but has larger biases for both *K*_↓,d_ (MBE = −22.95 W m^−2^) and *K*_↓_ (MBE = 16.85 W m^−2^).

### Limitations and Future work

In the present study, our objective was to compare a conceptual *k*_t_ model, a linear model, and an RF model that explicitly considers non-linear interactions to bias-adjust the MERRA-2 *K*_↓_ and *K*_↓,d_ fields. Based on the cross-validation, the RF model was used to develop the BaRAD dataset. It should be noted that there are many machine learning architectures that can capture non-linear interactions. A comprehensive cross-validation of all such models is beyond the scope of the present study but should be undertaken in future work. Compared to many of these other architectures, RF models are easier to train, less sensitive to hyperparameters, simple to interpret, and have been used in similar supervised learning problems with similar sample sizes^40^. Although we expect the improvements in bias-adjusted radiation fields to be minor when moving to more complicated machine learning models for the current training data, architectures like deep neural networks are expected to perform better as the training sample size increases. For larger sample sizes, feature selection would also be much more important to optimize training time and further improve accuracy.

Here we focus on monthly means since we have the most comprehensive geographic distribution of radiation observations at this temporal scale through GEBA. As more data are incorporated in this archive, we plan to update the BaRAD dataset. It is possible to generate datasets similar to BaRAD at sub-monthly and even sub-daily scales, though this requires more comprehensive training data than currently available. Observation networks like BSRN can help in this regard, but it is critical to set up new observation sites to continuously observe *K*_↓,d_ to reduce sampling biases, especially in tropical regions where *K*_↓,d_ would have a stronger influence on the terrestrial carbon, energy, and water cycles^23^.

## Usage Notes

The BaRAD dataset^35^ developed here performs well when compared to the GEBA dataset and captures the seasonal, latitudinal, and long-term trends in *K*_↓_ and *K*_↓,d_. However, the dataset can be affected by biased sampling in the GEBA dataset. The GEBA dataset is overrepresented in the northern hemisphere, especially in Europe and China^10,28^. A second source of bias is associated with the lack of training data over ocean surfaces. Finally, polar regions are under-sampled by GEBA as noted above. We urge caution when using this dataset over polar regions and ocean surfaces. For land grids in the southern hemisphere, although there are many observations for *K*_↓_, there are fewer stations with *K*_↓,d_ measurements. Even though Fig. 5 suggests that the BaRAD *K*_↓,d_ has less bias than the MERRA-2 dataset for sites not ‘seen’ by the bias-correction algorithm, when possible, we suggest independent validation of the BaRAD *K*_↓,d_ data before its applications for southern hemisphere land grids. For basic visualization, we have also developed a Google Earth Engine^41^ web application (https://yceo.users.earthengine.app/view/barad), that will allow one to download the time series of monthly diffuse and direct beam radiation for any grid. A summary of the datasets compared in the present study are given in Table S2. For a comprehensive comparison of reanalysis datasets that archive *K*_↓,d_, see Chakraborty & Lee^10^.

## Supplementary information

Supplementary Information

## Data Availability

The scripts used to generate the BaRAD dataset are available in this GitHub repository: https://github.com/TC25/BaRAD/tree/main/Scripts.
