# Supplementary material for: Using supervised learning to develop BaRAD, a 40-year monthly bias-adjusted global gridded radiation dataset
Source: Sci Data. 2021 Sep 15;8:238. doi: 10.1038/s41597-021-01016-4 (PMC8443559; doi:10.1038/s41597-021-01016-4)
Supplement: Supplementary file 1 — Supplementary Information [file 41597_2021_1016_MOESM1_ESM.pdf]

Title

Using supervised learning to develop BaRAD, a 40-year monthly bias-adjusted global gridded radiation dataset – supplementary information

Authors

TC Chakraborty[1], Xuhui Lee[1]

Affiliations

[1]School of the Environment, Yale University, New Haven, CT 06520, USA
corresponding author: TC Chakraborty (tc.chakraborty@yale.edu)

Table of Contents:

Figures S1 to S7 from pages 2 to 8

Tables S1 and S2 on pages 9 and 10

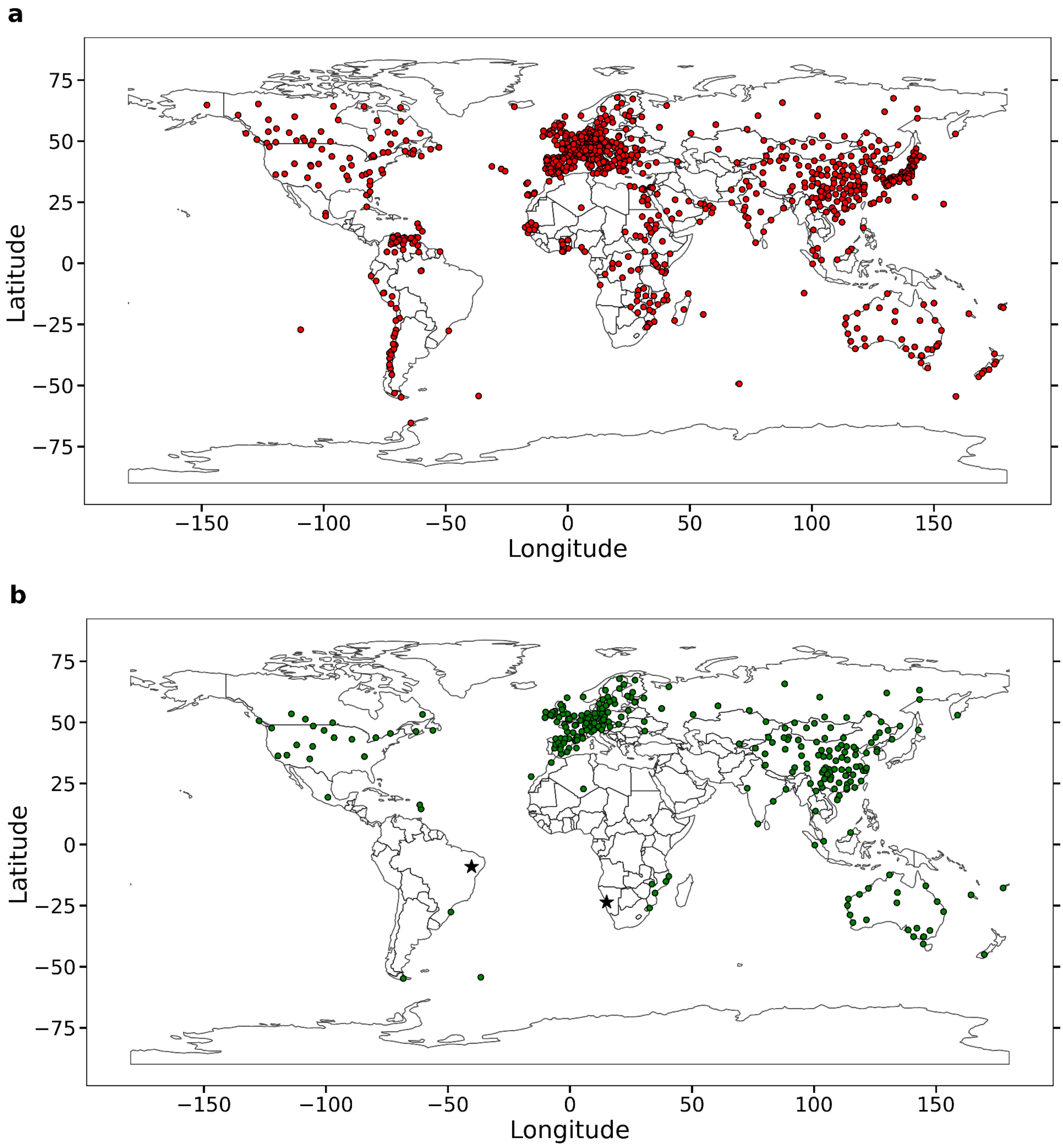

Figure S1: Spatial distribution of ground observations. Distribution of GEBA sites used for evaluating and training bias-correction algorithms in the present study for (a) shortwave radiation and (b) diffuse radiation. Sub-figure (b) also shows the locations (as black stars) of the two BSRN stations used to independently validate the BaRAD product.

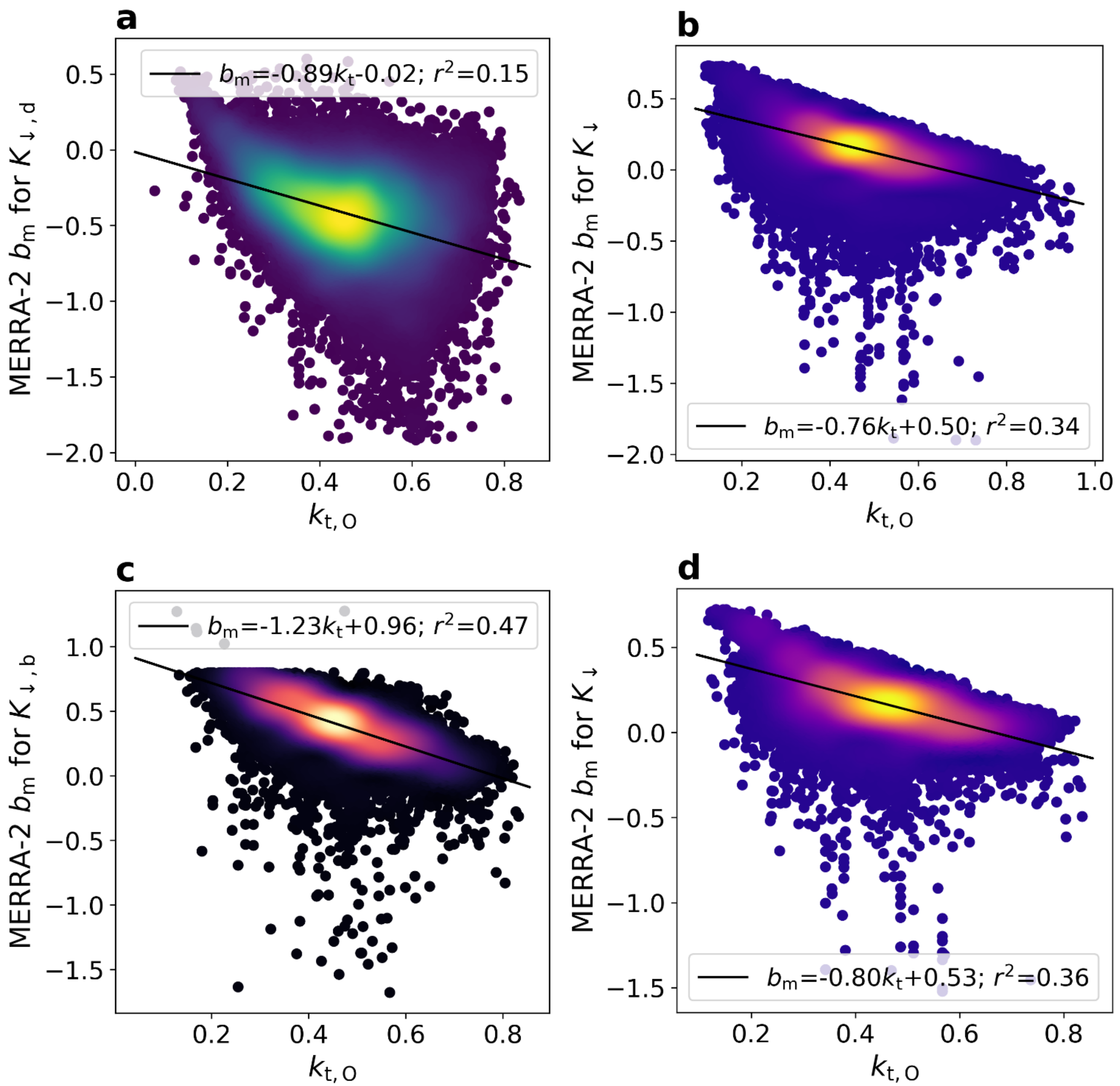

Figure S2: Control of clearness index on biases in the MERRA-2 dataset. (a) Bias in diffuse radiation ($K_{\downarrow,d}$), (b) bias in total shortwave radiation ($K_{\downarrow}$), (c) bias in direct beam radiation ($K_{\downarrow,b}$), and (d) bias total shortwave radiation ($K_{\downarrow}$) for the sites that also have $K_{\downarrow,b}$ measurements. Statistical summaries of the associations are noted. Color indicates data density.

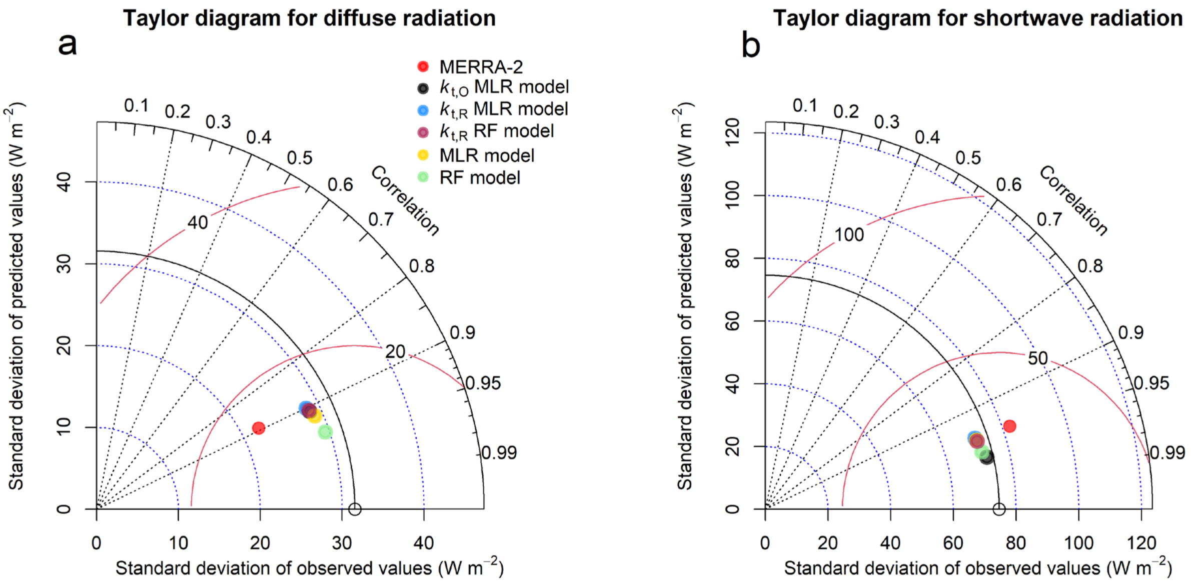

Figure S3: Taylor diagrams of bias-correction models. The Taylor diagrams represent the observed radiation values and predicted values from MERRA-2, the $k_{t,O}$ models, the $k_{t,R}$ models, the MLR models, and the RF models for the consolidated validation data for (a) $K_{\downarrow,d}$ and (b) $K_{\downarrow}$.

.

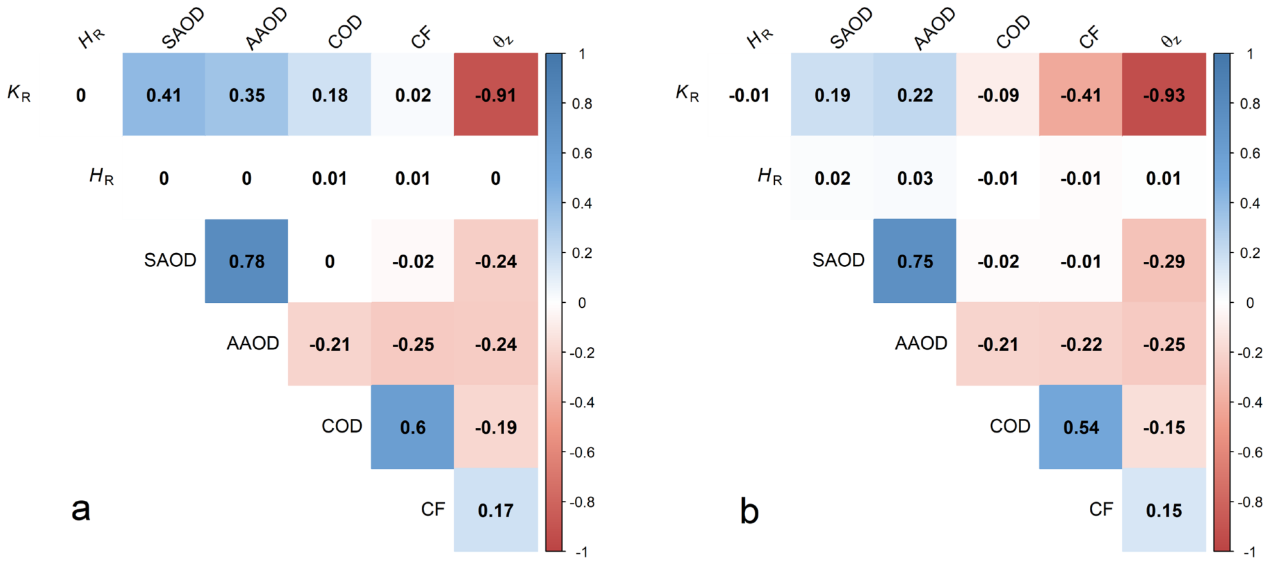

Figure S4: Correlation matrices of features. The correlation matrices of the features selected for training the supervised machine learning models for (a) $K_{\downarrow,\mathrm{d}}$ and (b) $K_{\downarrow}$.

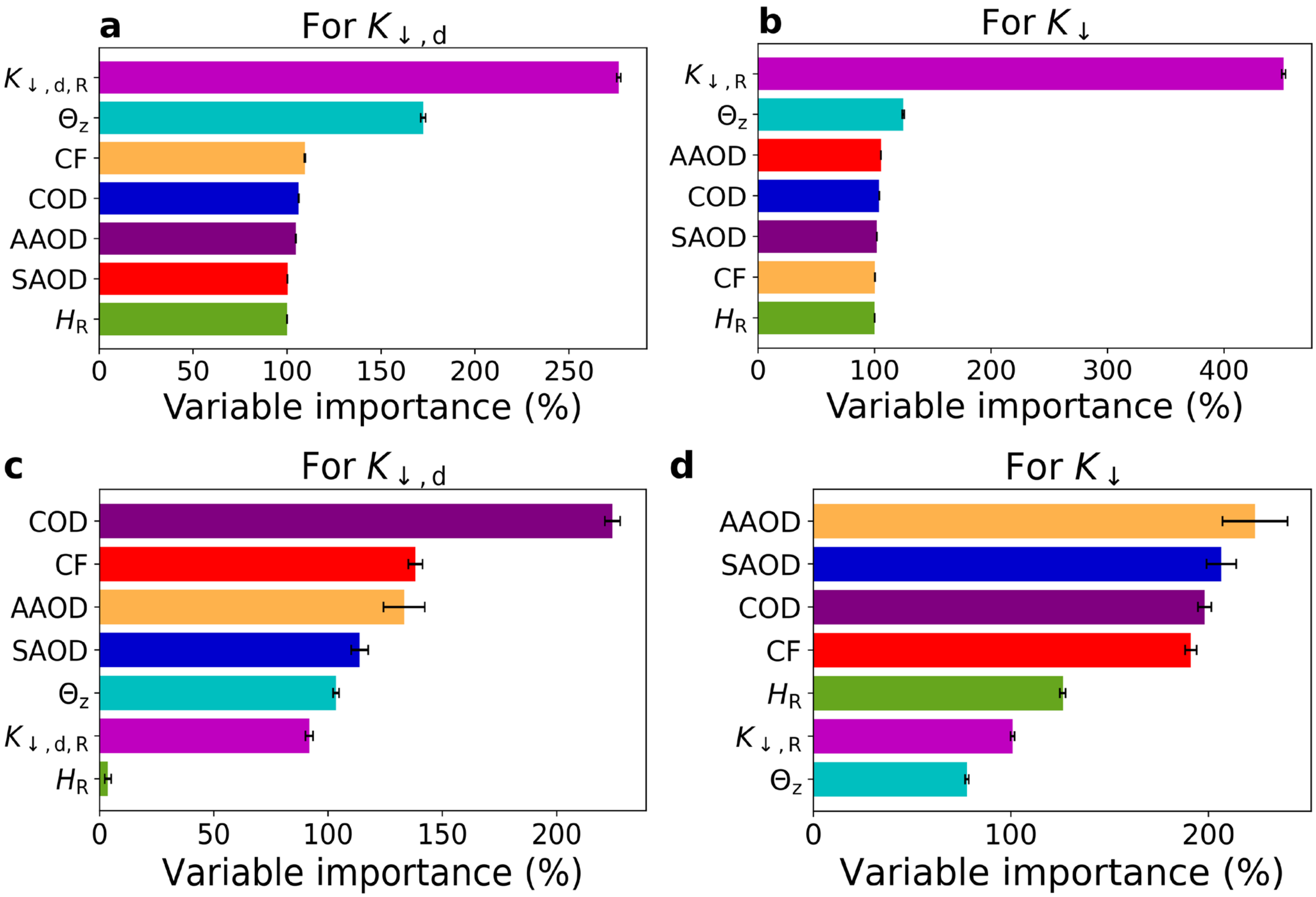

Figure S5: Rank of variable importance for bias-correction. (a) and (b): Permutation importance scores for the MLR model; (c) and (d): permutation importance scores for the RF model. The error bars show the standard deviation across the ten folds.

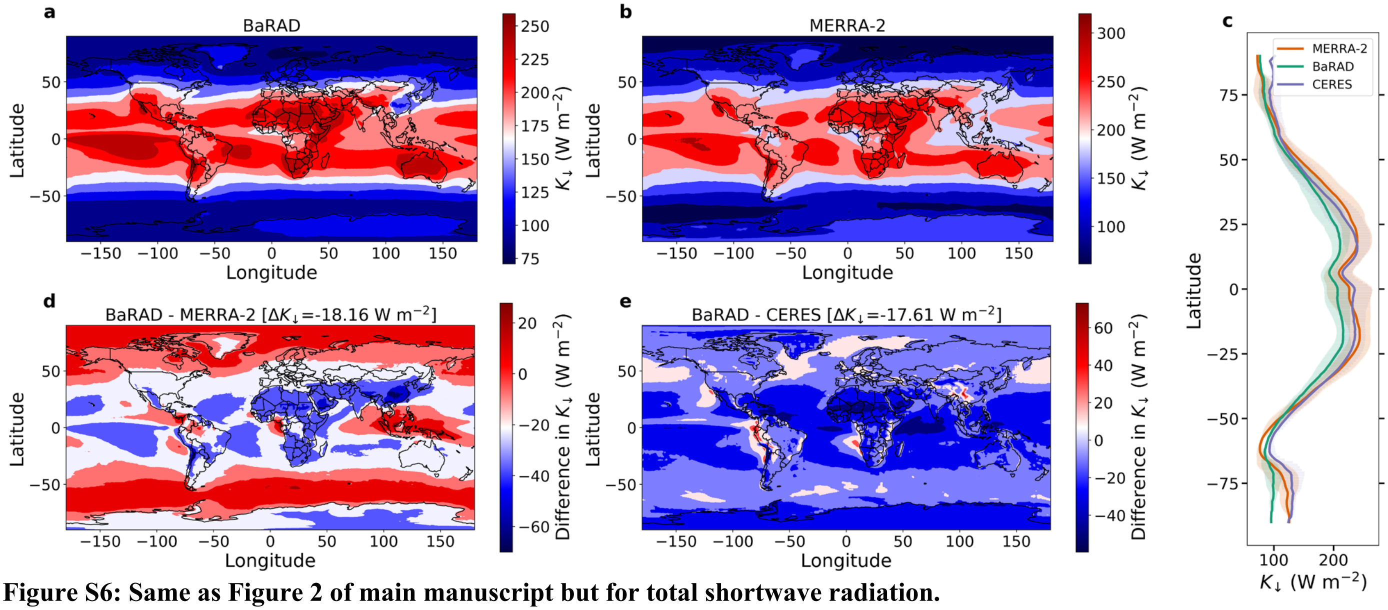

Figure S6: Same as Figure 2 of main manuscript but for total shortwave radiation.

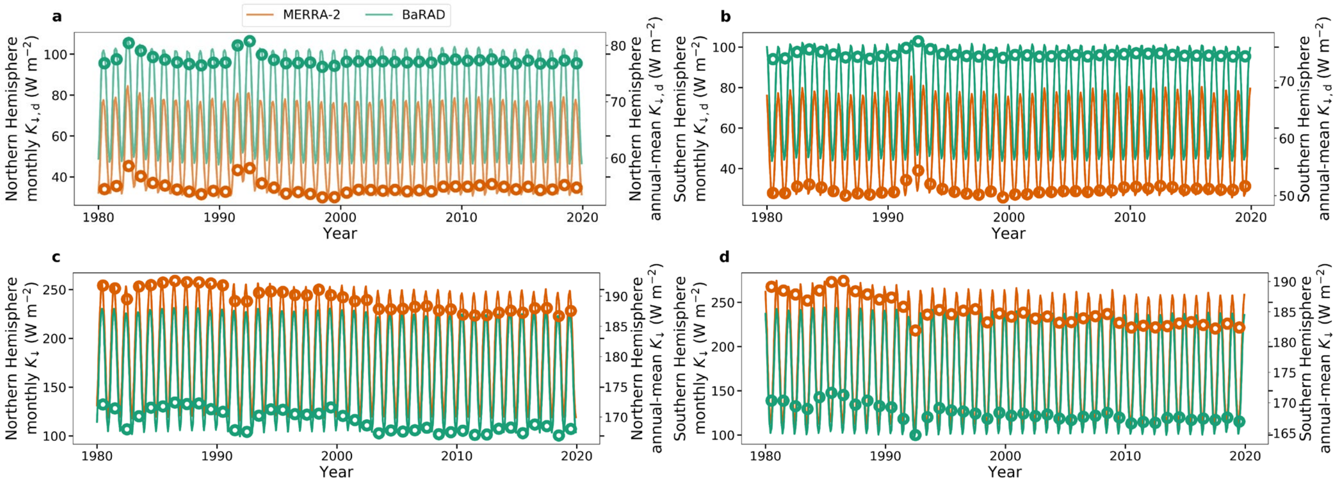

Figure S7: Long-term trends at regional scale. Sub-figures (a), (b), (c), and (d) show the long-term regional trends in $K_{\downarrow,\mathrm{d}}$ and $K_{\downarrow}$ for northern and southern hemispheres, respectively. The monthly values are plotted on the left y-axes as lines and the annual averages (plotted as circles) are on the right y-axes.

Table S1: Summary of features. Summary of the features, including their symbols and data source, used in the MLR and RF bias-correction algorithms.

Feature Name	Feature Symbol	Description	Data Source
Incoming radiation at surface	$K_{\downarrow, \mathrm{R}}$	Monthly grid-averaged value of incoming radiation at the surface. Can be either the total shortwave radiation reaching the surface ($K_{\downarrow}$), or its diffuse component ($K_{\downarrow,\mathrm{d}}$), which is the portion after the light is scattered.	MERRA-2 reanalysis
Scattering Aerosol Optical Depth	SAOD	Monthly grid-averaged optical depth of scattering aerosols in the atmospheric column.	MERRA-2 reanalysis
Absorbing Aerosol Optical Depth	AAOD	Monthly grid-averaged optical depth of absorbing aerosols in the atmospheric column.	MERRA-2 reanalysis
Cloud Optical Depth	COD	Monthly grid-averaged optical depth of all clouds in the atmospheric column.	MERRA-2 reanalysis
Cloud Fraction	CF	Monthly grid-averaged cloud fraction.	MERRA-2 reanalysis
Zenith Angle	θ_{z}	Monthly grid-averaged zenith angle, for the angle between the sun and the vertical direction.	Calculated
Altitude	H_{R}	Average altitude of the grid.	MERRA-2 reanalysis

Table S2: Summary of data products. List of data products included in the present study, along with their temporal and spatial resolution, and a few advantages and disadvantages

Dataset	Reference	Spatial Resolution	Finest temporal Resolution	Years of data availability	Advantages	Disadvantages
MERRA-2	24	0.5° x 0.625°	Hourly	1980 – Present	Physical model; Constrained by assimilated observations	Model parameterizations; Large biases in surface radiation
CERES	35	1° x 1°	Hourly	2001 – Present	Simplified radiative transfer model; Constrained by satellite observations	Model parameterizations; Large biases in surface radiation; Note available before 2001
DSCOVR/EPIC	21	0.1° x 0.1°	Hourly	June 2015 – June 2019	Data-driven model; Constrained by *in situ* and satellite observations	Limited period of availability; Not continuous at hourly scale; Data-driven
GEBA	28	Point	Monthly	Various; site-specific	Observations	Uneven geographic and temporal distribution; Sensor errors
BSRN	37	Point	Every Minute	Various; site-specific	Observations	Uneven geographic and temporal distribution; Sensor errors
BaRAD	Present study	0.5° x 0.625°	Monthly	1980 – 2019	Data-driven model; Constrained by *in situ* observations and MERRA-2 fields	Monthly scale; Data-driven; Influenced by sampling bias in training data
